# Pityriasis lichenoides chronica induced by COVID-19 messenger RNA vaccination

**DOI:** 10.1016/j.jdcr.2022.07.017

**Published:** 2022-07-19

**Authors:** Abdullah M. Al Muqrin, Ziyad Muharib N. Alruwaili

**Affiliations:** a Dermatology, Prince Mohammed Medical City, AlJowf, Saudi Arabia; b College of Medicine, Jouf University, AlJowf, Saudi Arabia

**Keywords:** COVID-19, pityriasis lichenoides, pityriasis lichenoides chronica, pityriasis lichenoides et varioliformis acuta, PLC, PLEVA, vaccination, PLC, pityriasis lichenoides chronica, PLEVA, pityriasis lichenoides et varioliformis acuta

## Introduction

Pityriasis lichenoides chronica (PLC) represents one end of the pityriasis lichenoides spectrum, which constitutes the chronic part of the spectrum, while the acute end of pityriasis lichenoides is represented by pityriasis lichenoides et varioliformis acuta (PLEVA).^1^ The pathogenesis of PLC and PLEVA has yet to be fully elucidated. However, there have been 3 postulated theories: an inflammatory response elicited by infectious agents, an inflammatory response due to T-cell dyscrasia, and a type III hypersensitivity reaction (immune complex–mediated vasculitis).^1^ Further, it has been linked to many possible inciting agents, such as *Toxoplasma gondii*, Epstein-Barr virus, human immunodeficiency virus, cytomegalovirus, and varicella-zoster virus, in addition to different types of vaccines.^1^, ^2^, ^3^, ^4^, ^5^, ^6^, ^7^, ^8^, ^9^, ^10^, ^11^ Vaccines reported as possible inciting triggers include vaccines for human papillomavirus, measles, mumps, rubella, tetanus, diphtheria, influenza, and COVID-19.^1^, ^2^, ^3^, ^4^, ^5^, ^6^, ^7^, ^8^, ^9^, ^10^, ^11^, ^12^ In this article, we present the second case report of PLC triggered by the Pfizer-BioNTech COVID-19 vaccine. We believe that this case report will reinforce the concept that COVID-19 vaccination could be a possible trigger and should be sought during history taking, especially during this era of COVID-19.

## Case report

**Fig 1:**
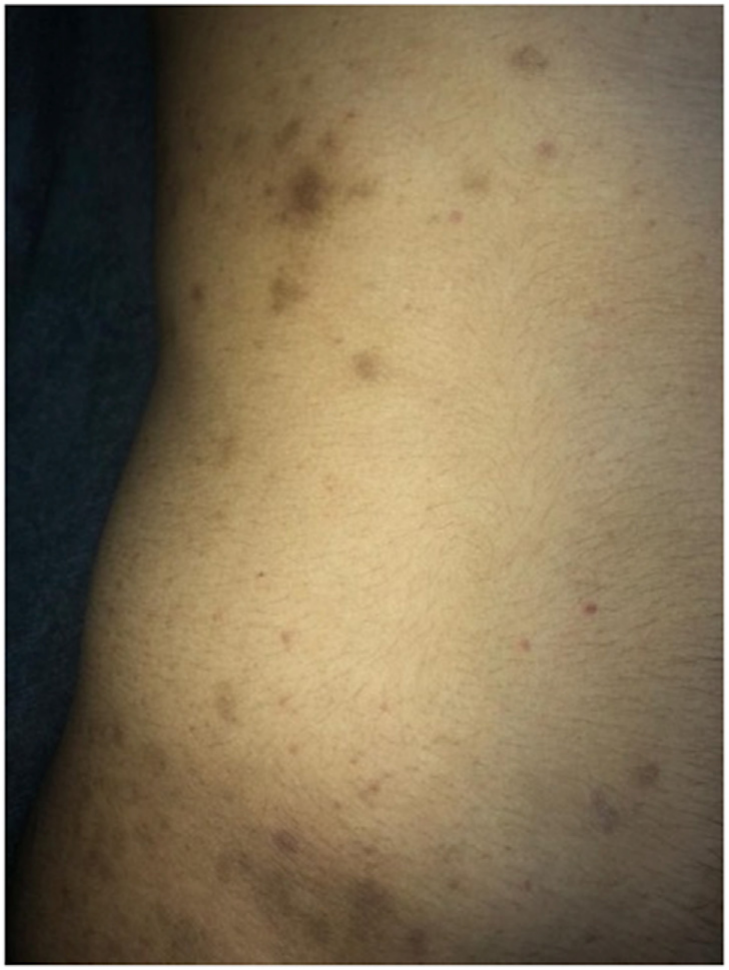
Multiple erythematous scaly papules admixed with postinflammatory hyperpigmentation.

**Fig 2:**
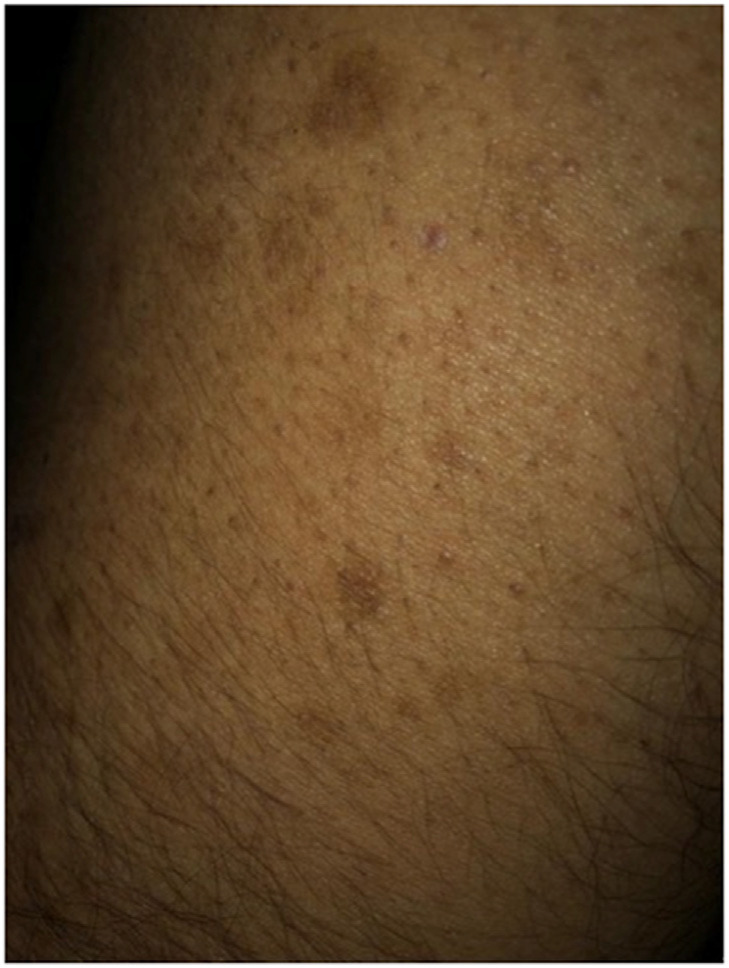
Solitary erythematous papules with multiple postinflammatory hyperpigmentation.

**Fig 3:**
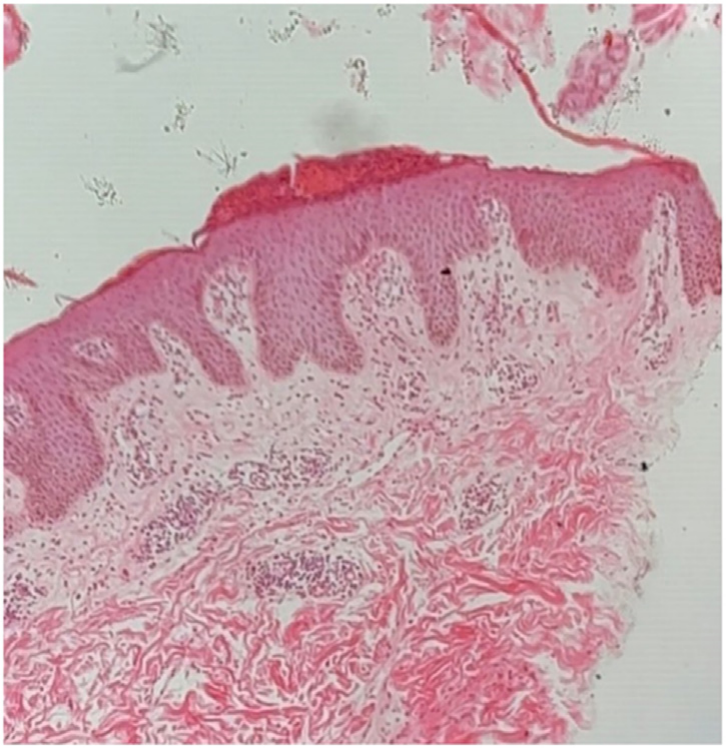
The low-power (H&E) stained histopathology slide shows focal parakeratosis, irregular acanthosis, and superficial dermal lymphocytic infiltrates. *H&E*, Hematoxylin and eosin.

**Fig 4:**
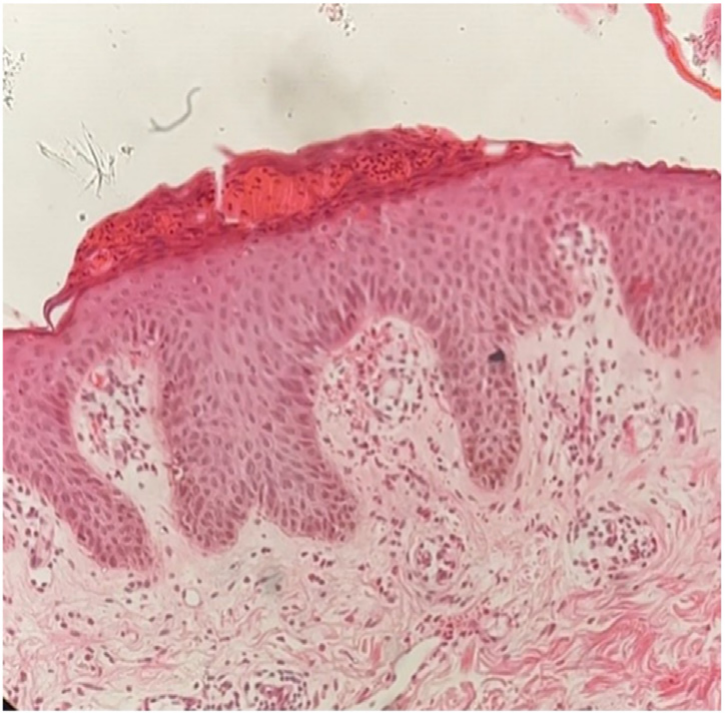
The high-power (H&E) stained histopathology slide reveals focal parakeratosis, acanthotic epidermis, and superficial lymphocytic infiltrates with extravasated *red* blood cells. *H&E*, Hematoxylin and eosin.

A 31-year-old Saudi man who was otherwise healthy presented to the dermatology clinic complaining of recurrent skin eruptions over the trunk and extremities for 10 to 15 days. The patient stated that this eruption was noted approximately a month after he received the first dose of the Pfizer-BioNTech COVID-19 vaccine. The rash was mild in severity until he received the second dose of the Pfizer-BioNTech COVID-19 vaccine, when he started developing more frequent recurrences of the rash within 10 to 15 days of the second dose. The patient denied any history of upper respiratory tract infections or drug injections before the eruption. He denied a history of fever or other systemic symptoms. Clinical examination revealed multiple erythematous scaly papules admixed with postinflammatory hyperpigmentation distributed over the trunk and proximal extremities (Figs 1 and 2). Mica-like scales were also observed in some of the lesions. Therefore, a punch skin biopsy was performed to confirm the diagnosis. Histopathological examination revealed focal parakeratosis, superficial dermal lymphocytic infiltrates, and focal areas of red blood cell extravasation (Figs 3 and 4). Based on clinical presentation and biopsy findings, a diagnosis of PLC was made, and the patient was prescribed doxycycline 100 mg twice daily.

## Discussion

The etiology of pityriasis lichenoides remains unknown. Many possible causative agents have been reported like infectious agents as well as some types of vaccines. Vaccination was first reported as a possible inciting agent of pityriasis lichenoides in 1992 by Torinuki, when he documented Mucha-Habermann disease induced by a freeze-dried live attenuated measles vaccine.^12^ Thereafter, several cases have been reported during the past decade regarding the possible association between vaccination and pityriasis lichenoides infection. To date, 12 cases have been reported in the literature linking different types of vaccines with pityriasis lichenoides (Table I). The most frequent vaccine types associated with the development of pityriasis lichenoides are measles, mumps, and rubella vaccines.^4^, ^5^, ^6^^,^^11^ Of the 12 published case reports, the most common pityriasis lichenoides type related to vaccination was PLEVA, constituting a total of 8 cases.^2^^,^^6^, ^7^, ^8^, ^9^, ^10^, ^11^^,^^13^ The remaining 4 cases presented with the following: 2 cases with PLC, 1 case with a mixture of both PLC and PLEVA, and the remaining case had Mucha-Habermann disease. In this report, we document the second case of PLC following the administration of the Pfizer-BioNTech COVID-19 vaccine. To better link the association between vaccination and PLC, we used the Adverse Drug Reaction Probability Scale (Naranjo Scale) and found that our case scored 7, which indicates a probable causality relationship between the Pfizer-BioNTech vaccine and PLC.^14^ However, it is important to highlight that the Naranjo Scale is a 10-question–based score system, and one of these questions mandates the presence of 2 or more published case reports with the same adverse events to count the score of that question.^14^ Therefore, this case could aid in the identification of the COVID-19–related PLC vaccine when the Naranjo scale is used, as it is the second case to be published in this regard.

**Table I:** Cases have been reported in the literature linking COVID-19 vaccines with pityriasis lichenoides

Reference	Age, y	Sex	Vaccine	Presentation	Latency period after the first dose (d)	Comorbidities
Dawoud et al^3^	16	Male	Pfizer-BioNTech	PLC	7	Unknown
Mäkilä et al^8^	21	Female	Pfizer-BioNTech (second dose)	PLEVA	10	Unknown
Palmén et al^10^	81	Male	Pfizer-BioNTech (first dose)	PLEVA	9	Unknown
Sechi et al^13^	70	Male	Pfizer-BioNTech (second dose)	PLEVA	5	Acute lymphocytic leukemia

*PLC*, Pityriasis lichenoides chronica; *PLEVA*, pityriasis lichenoides et varioliformis acuta.

## Conflicts of interest

None disclosed.
